# Anthropometric Measurements, Serum Reproductive Hormonal Levels and Sexual Development among Boys in the Rural Western Cape, South Africa

**DOI:** 10.3390/ijerph13121185

**Published:** 2016-11-29

**Authors:** Jun Mao, Mohamed Aqiel Dalvie

**Affiliations:** Centre for Environmental and Occupational Health Research, School of Public Health and Family Medicine, Health Sciences Faculty, University of Cape Town, Anzio Road, Observatory, Cape Town 7729, South Africa; maojunme@gmail.com

**Keywords:** growth, sexual development, reproductive hormones, boys, rural

## Abstract

Data on growth and sexual maturation among boys from the rural Western Cape in South Africa is limited. A cross-sectional study of 269 school boys was conducted testing for serum luteinizing hormone (LH), follicle stimulating hormone (FSH), testosterone, sex hormone binding globulin (SHBG) and estradiol (E2); height, weight and body mass index (BMI); sexual maturity (using Tanner Stages) and a questionnaire (demographic and medical history). The median age at pubertal onset (Tanner Stage 2) and Tanner Stage 5 was 11.6 and 14.7 years, respectively. The median testicular volume was 5.5 mL at Tanner Stage 2 increasing from 2.5 mL at Tanner Stage 1 to 14.7 mL at Tanner Stage 5. Height and weight measurements were <25th & 50th percentile references at Tanner Stages 1–3. Controlling for confounders, serum FSH and LH increased (*p* < 0.05) from Tanner Stage 1 to 4 (by 4.1 and 3 mL respectively), and serum testosterone and estradiol increased after Tanner Stage 2 (by 12.7 nmol/L and 59.5 pmol/L respectively). These results indicate some delays in pubertal development of boys in the rural Western Cape when compared to boys from other settings possibly due to nutritional, socio-economic and environmental exposures. Changes in serum hormone levels were consistent with other populations. Initiatives to improve nutrition amongst Western Cape rural communities are recommended.

## 1. Introduction

Puberty involves a number of processes, including the adolescent growth spurt, rapid changes in body composition, development of secondary sex characteristics, activation of hypothalamic–pituitary–gonadal axis activity, achievement of fertility, and behavioral and psychological changes. Physical development during puberty can be measured by assessing secondary sex characteristics, bone age, growth spurts, or hormonal levels with each measurement capturing a different aspect of the pubertal process and none of these representing the “gold-standard” [1].

The Centers for Disease Control and Prevention (CDC) and the World Health Organization (WHO) Growth Standards are the most well-known growth standards for anthropometric measurements (height, weight, body mass index), and are used internationally to monitor growth and identify potential health- or nutrition-related problems among children and adolescents [2]. The growth of nutritionally disadvantaged children is generally characterized by a short stature and low weight, as well as late biological maturation [3,4,5]. On the other hand, pubertal growth not only determines final height in adulthood, but also may serve as a catch-up period to regain the previous growth loss [3,6,7,8]. It is therefore important to have local growth reference ranges by sexual maturation from developing regions.

The development of secondary sex characteristics is driven by the activity of reproductive hormones in the hypothalamic–pituitary–gonadal axis (HPG) and is commonly measured by five Tanner Stages [9]. The HPG axis which is the major regulator of the mature reproductive system, is quiescent in childhood and then reactivated at the onset of puberty [10]. This reactivation of the HPG axis is characterized by a sudden increase in the concentration of gonadotropins follicle stimulating hormone (FSH) and luteinizing hormone (LH) stimulated by gonado-trophin-releasing hormone (GnRH) in the pituitary gland, and sex steroids (testosterone and oestradiol) stimulated by gonadotropins in the gonads [11].

FSH and inhibin B released by Sertoli cells after puberty and which functions as a negative feedback on FSH secretion, are useful markers of spermatogenesis and Sertoli cell function reflecting the quality of semen, particularly semen concentration [12,13,14]. A Danish study of 430 couples with unknown fertility found 100% predictive power for detecting sperm counts below 20 mill/mL by using the criteria of basal inhibin <80 pg/mL and FSH >10 miu/mL [15]. A recent cross-sectional study of 47 DDT exposed malaria vector control workers reported a positive relationship (Odds Ratio = 37, Confidence Interval (CI): 2–655) between high basal estradiol (>50 pg/mL) and abnormal semen morphology (proportion < 5%) and low motility (proportion < 50%) indicating that estradiol could also be a reproductive marker [16].

Puberty is a critical development period for the establishment of a mature reproductive system as well as the achievement of adult levels of reproductive hormones. Therefore, monitoring the changes in reproductive hormones during puberty may provide an indication of the degree of reproductive development in boys and their fertility later in life.

Data on anthropometric characteristics, reproductive hormones and sexual development in pubertal children in the rural Western Cape of South Africa have, to our knowledge, not been published before. Considering the multitude of social impacts due to poverty as well as environmental impacts, there is a need for such local reference data among these populations. There are also few studies available in the literature and none in South Africa that have investigated the changes in serum reproductive hormone levels during pubertal development of children [11,17,18,19].

In this analysis, we present cross-sectional descriptive data on anthropometric characteristics, secondary sexual characteristics, testicular volumes and reproductive hormones from boys residing in the rural Western Cape in South Africa participating in a study that has investigated the effects of pesticide exposure on these populations. Additionally, the relationship between these parameters has been investigated.

## 2. Methods

### 2.1. Study Design, Population and Sampling

Data from a cross-sectional study of Western Cape rural boys in South Africa conducted between April 2007 and March 2008, was used in this analysis. The sampling for the study has been described in detail elsewhere [20]. The eight most accessible primary and secondary schools from three agricultural areas (Hex River Valley, Grabouw, Piketberg) and their neighboring non-agricultural rural areas in the Western Cape Province were chosen for the study. School boys aged from 5 to 19 years were chosen in order to cover the full age range for pubertal development.

Four hundred and ninety-two boys’ parents consented to participate in the study and were stratified according to whether they had lived on a farm or not at the time of the study and by area. Then 274 boys were selected including all boys (*n* = 180) living on a farm and 94 not living on a farms. The former group was chosen by random systematic sampling, stratified equally by age groups. A further 5 boys all who lived on a farm did not participate in the study leaving only 269 participants. In the study sample, 15.2% ranged between 5 to 9 years of age (pre-pubertal), 28.6% ranged between 9.1 to 11 years of age (early-puberty), 44.2% ranged between 11.1 to 14 years of age (mid-puberty) and 12% exceeded 14 years of age (post-puberty).

### 2.2. Measurements

A questionnaire was administered to a parent of the participant by trained fieldworkers using mobile technology. The questionnaire was developed by the study team, led by the principal investigator, and was based on previous local studies in similar populations [21,22]. The questionnaire included sections on demography, genital health history, and general medical history.

Anthropometric measurements and sexual maturity assessment was conducted by a male nurse who was trained by a local reproductive health specialist. Training was conducted over a period of 2 days, mainly focusing on demonstrating how to perform anthropometric measurements, use of the orchidometer to measure testicular volume and assessing the sexual maturity rating (SMR) by examination of boy patients and using visual material.

Height and weight were recorded using a calibrated scale according to standardized methods, and body mass index (BMI) was calculated. The Sexual Maturation Rating was recorded according to 5 Tanner Stages [23]. Testicular volume was assessed using a standardized set of wooden testicular beads [24], and further examinations were conducted to assess for the presence of genital scars, penis and testicular abnormalities such as congenital hydroceles, undescended testes, congenital inguinal hernias and hypospadias.

Blood samples (12 mL) were collected between morning and early-afternoon. The blood samples were centrifuged at 5000 revolutions per minute in the field and the serum stored and transported on ice to National Health Laboratory Sciences (NHLS) facility at Groote Schuur Hospital in Cape Town within 24 h. LH, FSH, testosterone, oestradiol, and SHBG were measured using electrochemiluminescence immunoassays (ECLIA) on a Roche Cobas Modular E170 analyzer (Roche Diagnostics, Indianapolis, IN, USA).

This study was done according to the Declaration of Helsinki of the 25th World Medical Assembly [25]. The research proposal of the study was approved by the University of Cape Town’s Research Ethics Committee (HREC REF 943/2014). The study respected participants’ autonomy, the right to the information and confidentiality of their information.

### 2.3. Statistical Analysis

Data analysis was performed using Stata 12 statistical software (StataCorp, College Station, TX, USA). Exploratory data analysis was conducted first. Each variable was examined for missing data, and then assessed by univariate graphs and summary statistics for any potential outliers. Normality for continuous variables was assessed using histograms and Shapiro-Wilk tests. Descriptive statistics were calculated and expressed as medians and interquartile intervals.

To compare with CDC growth charts and WHO growth reference [26,27], two binary variables for each variable such as height, weight and BMI were generated by using the 25th and 50th percentile (≤25th and 50th percentile = 1, ≥25th and 50th percentile = 0) for age from both growth references as cut-offs.

Bivariate analysis was conducted to explore the relationships between independent variables (age, anthropometric variables, testicular volume and hormones) and possible predictors to identify potential confounders. Household income, birth weight and SHBG were tested by the Pearson’s correlation test (for normally distributed data) and the Spearman’s Rank correlation test (for non-normally distributed data) with all predictor variables. Residential location, general health, disease history and testicular related problems were tested by *t*-test (for normally distributed data) and Wilcoxon rank sum test (for non-normally distributed data) with all predictor variables. Confounders were selected for multivariate model building if the association was *p* < 0.1. Linear regression analysis was used for investigating the multivariate association between dependent and predictor variables by Tanner Stage (Dummy variables were generated from the 5 discrete Tanner Stage variables including Tanner Stage 1 vs. Tanner Stage 2, Tanner Stage 2 vs. Tanner Stage 3, Tanner Stage 3 vs. Tanner Stage 4; Tanner Stage 4 vs. Tanner Stage 5). Model building was started with an empty model containing only the constant and then adding predictors. Forward Stepwise Regression procedure was used to select the best combination of confounders. The likelihood ratio test was used to determine whether the model has been significantly improved by adding each confounder. The variable combination with lowest Aikake’s Information Criterion (AIC) statistic was selected for the next step until the accomplishment of the best combination of variables and then the key independent variable to the final model. Regression diagnostics were determined by the goodness of fit of the model, and outliers or influential observations were assessed.

## 3. Results

### 3.1. Participation, Demographic Characteristics, Socioeconomics Information and Health Status and Medical History

One hundred and seventy-seven of the participating boys (65.8%) lived or previously lived on a farm and 92 boys (34.2%) lived only in a town. Overall the median age of the participants was 11.6 years (Table 1) and the median birthweight was 2.9 kg (*n* = 233). The median household income (*n* = 262) was R 2000 per month. The sexual maturity assessment of the boys found that (Table 1) 39.78% (*n* = 107) of boys were classified in the pre-pubertal stage, 43.5% (*n* = 117) in the mid-puberty stage (Tanner Stages 2 and 3), and 16.35% (*n* = 44) in late puberty (Tanner Stages 4 and 5). A small proportion of parents (3.7%) reported that their participating children were in a poor health condition. Lifetime tuberculosis and asthma reported was more than 5%, about and a third (29.3%) previously had mumps. About 5% of participants were born with abnormal testis and less than 3% had a previous testicular injury or operation or disease.

### 3.2. Testicular Development and Age of Onset of Sex Maturational Development

Age and testicular volumes were significantly (*p* < 0.05) positively associated with the increase in Tanner Stage until Tanner Stage 4 (Table 2).

### 3.3. Relationship between Anthropometric Variables and Sexual Development

A description of anthropometric characteristics, sexual development, and reproductive hormone levels have been presented in English et al. (2012) [20], and are presented in Table 2 by Tanner Stage. All anthropometric variables increased during the development of puberty.

Multivariate analysis between Tanner Stage and anthropometric variables in Table 3 shows that height and weight all increased significantly (*p* < 0.05) by Tanner Stage but height did not increase significantly between Tanner Stage 4 and Tanner Stage 5. The only significant increase in BMI was between Tanner Stages 2 and 3, with the regression coefficient projecting an increase of 2.27 kg^2^/cm (95% confident interval between 1.13 and 3.414 kg^2^/cm). Coincidently, the most significantly growth in height and weight was also found in between Tanner Stages 2 and 3, with the regression coefficient projecting an increase of 13.8 cm in height, and 11.9 kg increase in weight. This indicated that the most rapid period of pubertal growth lies between Tanner Stage 2 and stage 3 which corresponds to an age of 11.6 to 13.1 years among the Western Cape rural boys.

When comparing the age specific height and weight with CDC growth charts and WHO growth standards more than 25% and 50% of Western Cape rural boys were below the 25th and 50th percentile respectively of both WHO and CDC growth charts during the pre- and early-puberty (Table 4). After early puberty (Tanner Stage 3), the height and weight measurements caught up with CDC and WHO standards. Slightly less boys were below WHO charts than CDC charts.

### 3.4. Serum Reproductive Hormones by Tanner Stage

The multivariate relationship between Tanner Stage and reproductive hormones are shown in Table 5. Serum FSH and LH increased significantly (*p* < 0.001) from Tanner Stage 1 to Tanner Stage 4, and then reduced non-significantly until the end of puberty. Between Tanner Stage 1 to Tanner Stage 4, each increase in the Tanner Stage was associated with 1.034 IU/L to 1.821 IU/L increase in the serum levels of FSH, and 0.545 IU/L to 1.163 IU/L increase in LH. Serum levels of testosterone and oestradiol was increased non-significantly from Tanner Stage 1 to Tanner Stage 2, and then increased significantly (*p* < 0.05) thereafter. The increase in levels of testosterone and oestradiol were highest during Tanner Stages 4 and 5. In contrast, the serum levels of SHBG started decreasing significantly at the onset of puberty until Tanner Stage 4, and then remained constant to the end of puberty. When adjusting for SHBG, there were no significant increases in serum levels of LH and FSH during puberty (results not shown).

## 4. Discussion

This study found that the median age at the onset of puberty (Tanner Stage 2) of Western Cape rural boys to be 11.5 years, which was delayed in comparison to boys from settings with higher socio-economic status such as African American boys (9.5 years old) and American Caucasian boys (10.1 years old) from a US national probabilistic sampling study conducted between 1988 and 1994 [28]. Additionally, urban African boys (10.4 years old) and Caucasian boys (9.8 years old) from a birth cohort in Soweto, South Africa in 2004 [29], urban boys from Nairobi, Kenya (9.7 years old) in a study conducted in 1980 [3], and urban boys whose parents were employed in the formal sector from Choma, Zambia (11.2 years old) participating in a study conducted in 1993 [8] had an earlier median age at the onset of puberty than boys in this study. The late onset of puberty in Western Cape rural boys compared to African boys from developed countries and urban settings in Africa may be due to a lower nutritional status resulting from a lower socio-economic status of rural Western Cape boys. The study conducted in Zambia used a testicular volume of 3 mL as the cut-off value for onset of puberty in boys [8], while the rest of the studies used Tanner Genital Stage 2 as the onset of puberty [28,29].

Despite the delay in the age at onset of puberty, the median age at Tanner Stages 4 and 5 (13.9 and 14.7 years respectively) in Western Cape rural boys was similar to those of boys in Kenya and African American boys [28]. This may suggest that the duration of puberty in Western Cape rural boys is shorter than those of African populations with higher socio-economic status resulting from a catch-up in pubertal development similar and may be related to the catch-up in the anthropometric development as will be discussed in a later section. It should be noted that these results on the duration of pubertal development could have been affected by the smaller sample of boys over the age of 14 years in the study (12%) compared to the younger participants.

The low anthropometric readings of Western Cape rural boys when compared to CDC and WHO growth standards before Tanner Stage 3 and subsequent catch-up during Tanner Stages 4 and 5 could likely be attributed to poor nutritional conditions and adverse exposures also identified in other populations with stunting [3,5,30]. Puberty was identified as a catch-up period to make up the growth losses in animal experiments on re-feeding after undernutrition [31,32,33]. In epidemiologic studies comparing impoverished children to privileged children for both sexes [3,8,34], catch-up growth in children that mature later have been attributed to improved nutrition due to preferential feeding in these children and due to the fact that these children being more independent in sourcing food [3,35]. Another interesting finding of this study is that pubertal delay in boys with a generally lower BMI and a lower initial height does not prevent catch up development, with comparable final height and comparable reproductive hormones by the end of pubertal maturation, to those of previously described cohorts with high socio-economic standards.

This study found that LH and FSH in Western Cape rural boys increased substantially during Tanner Stages 1–4 and oestradiol and testosterone increased between Tanner Stages 2–5. Table 6 and Table 7 compare the levels of gonadotropins and sex steroids in this study to other published studies which are all from European countries [11,17,18,19] as no data could be found from other settings. It should also be noted that two of the previous studies [17,18] are longitudinal studies while the other two are cross-sectional. For both LH and FSH, the pattern of increase and the levels of hormones are similar to those of European boys apart from the fact that in Danish boys [11], FSH increase until Tanner Stage 3 and then stabilizes. The FSH levels at Tanner Stage 4 in the current study are also higher than those in previous studies. For testosterone, the levels are lower but the patterns of increase are similar to that of European boys. The generally high levels of testosterone in the study by Anderson et al. [11], might be a reason for the stabilization of FSH levels at Tanner Stage 3 resulting from negative feedback on the hypothalamus that lead to the lowering of GnRH and gonotrophins levels. The low levels of testosterone among Western Cape rural boys especially during Tanner Stage 1 and Tanner Stage 2 when compared to the European studies might have played a role in the delays in early puberty as testosterone stimulates secondary sexual characteristics in males. Only one previous study had investigated oestradiol in which the pattern of increase is similar to this study but the levels of oestradiol are lower [11].

Previous studies have found serum FSH to be negatively associated with semen quality and a marker of semen quality in men [12,15]. High FSH levels at Tanner Stage 4 in boys from the rural Western Cape were found in comparison to those in European boys listed in Table 6. Previous studies also found serum FSH and inhibin B to be associated with the number of Sertoli cells which determine testicular size and daily sperm production in men [36]. During puberty immature proliferating Sertoli cells switches to mature non-proliferating Sertoli cells, which determines the final number of Sertoli cells in adulthood [36,37], and this could explain the stabilization of the testicular volume apubertal Stage 4.

As mentioned before, the lower number of boys above 14 years of age group (12%) lowered statistical power for this group which might have been a limitation in the study. Another limitation in this study also noted before is that serum inhibin B which was found to be a strong marker of Sertoli cell function and spermatogenesis compared to FSH in previous studies, was not measured in this study. The comparisons of the results in the different studies are also limited by the different methods used in the laboratories and different definitions of puberty. Additionally, the cross-sectional design only captures a single time point during pubertal development of the boys and a longitudinal design whereby each boy is followed during pubertal development would be more accurate.

## 5. Conclusions

The age at onset of puberty as indicated by Tanner genital Stage 2 in Western Cape rural boys was late compared to boys from other settings. When late puberty approached, the delay in sexual maturation was diminished. A similar catch-up pattern was found in the height and weight increase, which were lower than CDC and WHO standards until Tanner Stage 3 and then normalized for higher Tanner Stages. The relatively low levels of testosterone in Western Cape rural boys may explain the delay in early maturational process. The concerns in the delay in anthropometric and sexual maturation during the early puberty amongst boys in these communities, may also impact on reproductive ability in adulthood. Initiatives to improve nutrition in Western Cape rural communities are recommended.

## Figures and Tables

**Table 1:** Participation, demographic characteristics, socioeconomics information.

Variables	Median (Interquartile Range)
Age (years), (*n* = 269)	11.58 (9.42; 13.17)
Monthly Household Income (Rands), (*n* = 262)	2000 (1300; 2720)
Birth weight (kg), (*n* = 233)	2.9 (2.51; 3.3)
**Variables**	***N* (%)**
Lifetime Residential Location	
Farm	177 (65.80%)
Non-farm	92 (34.20%)
Number of Boys within each Tanner Stage	
Tanner Stage 1	107 (39.78%)
Tanner Stage 2	78 (29.00%)
Tanner Stage 3	39 (14.50%)
Tanner Stage 4	36 (13.38%)
Tanner Stage 5	8 (2.97%)
General Health	
Good to Excellent	259 (96.3%)
Poor	10 (3.7%)
Medical History (Lifetime)	
Diabetes	2 (0.7%)
Tuberculosis	15 (5.6%)
Epilepsy	3 (1.1%)
Asthma	25 (9.3%)
Heart Problem	2 (0.7%)
HIV	1 (0.4%)
Foetal Alcohol Syndrome	2 (0.7%)
Mumps	79 (29.3%)
Pesticide poisoning	2 (0.7%)
Testicular Related Problems	
Born with abnormal testes	12 (4.5%)
Previous testicular injury	8 (3.0%)
Previous testicular operation	5 (1.9%)
Reported testicular disease	7 (2.6%)

**Table 2:** Mean and interquartile range of age, testicular volume, anthropometric variables and reproductive hormone levels per tanner stage amongst boys in the rural Western Cape.

Tanner Stage Variable	Tanner Stage 1	Tanner Stage 2	Tanner Stage 3	Tanner Stage 4	Tanner Stage 5
Age (Year)	9.3 (8.8; 10.7)	11.7 * (10.4; 12.8)	13.1 * (12.5; 13.9)	13.9 * (13.0; 15.0)	14.7 (12.9; 15.5)
Testicular Volume (mL)	2.5 (2.5–3.5)	5.5 * (4.0–9.0)	15.0 * (11.0–17.5)	20.0 * (17.5–22.5)	22.5 (17.5–22.5)
Anthropometric Variables					
Height (cm)	128.5 (124.0–135.0)	137.2 * (131.2–141.5)	149.7 * (144.4–159.0)	162.0 * (153.8–166.5)	165.5 (165.1–174.0)
Weight (kg)	27.0 (24.0–32.0)	32.0 * (29.0–37.0)	42.0 * (39.0–48.0)	49.0 * (45.0–54.5)	57.0 * (51.5–64.0)
BMI (kg^2^/cm)	16.64 (15.23–18.12)	17.25 (15.55–18.30)	18.50 * (17.23–22.21)	18.77 (17.76–20.64)	20.90 (19.17–23.44)
Reproductive Hormones					
FSH (IU/L)	0.9 (0.5–1.5)	1.9 * (1.3–3.1)	3.1 * (2.3–4.9)	5.0 * (3.2–7.5)	3.4 (3.2–7.0)
LH (IU/L)	0.05 (0.05–0.2)	0.7 * (0.2–1.0)	1.65 * (1.3–2.1)	3.0 * (1.8–4.2)	2.8 (1.9–3.8)
Testosterone (nmol/L)	0.05 (0.05–0.1)	0.2 (0.05–0.6)	3.9 * (1.6–8.4)	10.45 * (5.4–13.6)	12.9 * (10–18.9)
Oestradiol (pmol/L)	31.5 (23.1–45.5)	40.45 (29.7–50.4)	56.5 * (46.1–72.2)	79.35 * (61.5–88.5)	100.9 * (87.2–115.2)
SHBG (nmol/L)	121.8 (92.9–145.6)	104.8 * (86.7–129.6)	67.5 * (54–78.7)	44.9 * (37.6–50.2)	46.2 (36.6–55.2)

* *p* < 0.05 compared to the previous Tanner Stage (Linear Regression Analysis). BMI: Body mass index; FSH: follicle stimulating hormone; LH: luteinizing hormone; SHBG: sex hormone binding globulin.

**Table 3:** Multivariate association between anthropometric variables and tanner stage for boys from the rural Western Cape after adjusting for lived on farm or not, family income, birth weight, general health condition, prenatal exposures, and testicular related problems.

Outcomes	*N*	*p*-Value	B-Coefficient	95% Confidence Interval
Height				
Tanner Stage 2 vs. Stage 1	185	<0.001	7.536	(4.871; 10.201)
Tanner Stage 3 vs. Stage 2	117	<0.001	13.804	(10.122; 17.486)
Tanner Stage 4 vs. Stage 3	75	<0.001	8.576	(3.931; 13.222)
Tanner Stage 5 vs. Stage 4	44	0.296	3.725	(−3.280; 10.729)
Weight				
Tanner Stage 2 vs. Stage 1	185	<0.001	3.956	(1.796; 6.115)
Tanner Stage 3 vs. Stage 2	117	<0.001	11.911	(8.927; 14.895)
Tanner Stage 4 vs. Stage 3	75	0.014	4.737	(0.972; 8.502)
Tanner Stage 5 vs. Stage 4	44	0.012	7.295	(1.618; 12.972)
BMI				
Tanner Stage 2 vs. Stage 1	185	0.446	0.321	(−0.507; 1.149)
Tanner Stage 3 vs. Stage 2	117	<0.001	2.270	(1.126; 3.414)
Tanner Stage 4 vs. Stage 3	75	0.706	−0.276	(−1.720; 1.167)
Tanner Stage 5 vs. Stage 4	44	0.109	1.775	(−0.401; 3.951)

**Table 4:** Comparison of anthropometric measurements with CDC and WHO charts.

Height, Weight and BMI < 25th & 50th Percentile over Each Tanner Stage	Vs. CDC Growth Chart *n* (%)	Vs. WHO Growth Chart *n* (%)
Body Mass Index		
≤50th percentile for age	138 (51.49%)	116 (43.28%)
Tanner Stage 1	54 (50.47%)	44 (41.12%)
Tanner Stage 2	46 (58.97%)	42 (53.85%)
Tanner Stage 3	16 (41.03%)	14 (35.90%)
Tanner Stage 4	19 (52.78%)	13 (36.11%)
Tanner Stage 5	3 (37.50%)	3 (37.50%)
≤25th percentile for age	74 (27.61%)	64 (23.88%)
Tanner Stage 1	32 (29.91%)	29 (27.10%)
Tanner Stage 2	27 (34.62%)	24 (30.77%)
Tanner Stage 3	7 (17.95%)	6 (15.38%)
Tanner Stage 4	7 (19.44%)	5 (13.89%)
Tanner Stage 5	1 (12.50%)	0 (0.00%)
Height		
≤50th percentile for age	194 (72.39%)	179 (66.30%)
Tanner Stage 1	83 (77.57%)	76 (70.37%)
Tanner Stage 2	64 (82.05%)	60 (76.92%)
Tanner Stage 3	25 (64.10%)	22 (56.41%)
Tanner Stage 4	17 (47.22%)	16 (44.44%)
Tanner Stage 5	5 (62.50%)	4 (50.00%)
≤25th percentile for age	152 (56.72%)	136 (50.37%)
Tanner Stage 1	66 (61.68%)	59 (54.63%)
Tanner Stage 2	54 (69.23%)	47 (60.26%)
Tanner Stage 3	19 (48.72%)	17 (43.59%)
Tanner Stage 4	10 (27.78%)	9 (25.00%)
Tanner Stage 5	3 (37.50%)	3 (37.50%)
Weight		
≤50th percentile for age	179 (66.54%)	
Tanner Stage 1	74 (69.16%)	
Tanner Stage 2	65 (83.33%)	
Tanner Stage 3	22 (56.41%)	
Tanner Stage 4	15 (41.67%)	
Tanner Stage 5	3 (37.50%)	
≤25th percentile for age	112 (41.64%)	
Tanner Stage 1	50 (46.73%)	
Tanner Stage 2	43 (55.13%)	
Tanner Stage 3	11 (28.21%)	
Tanner Stage 4	8 (22.22%)	
Tanner Stage 5	0 (0.00%)	

**Table 5:** Multivariate Linear Regression relationship between tanner stage and reproductive hormone levels for boys in the rural Western Cape after adjusting for lived on farm or not, family income, general health condition, and ever had any testicular related problems.

Outcomes	*N*	*p*-Value	B-Coefficient	95% Confidence Interval
FSH				
Tanner Stage 2 vs. Stage 1	170	<0.001	1.034	(0.554; 1.514)
Tanner Stage 3 vs. Stage 2	110	<0.001	1.416	(0.765; 2.067)
Tanner Stage 4 vs. Stage 3	72	<0.001	1.821	(0.986; 2.657)
Tanner Stage 5 vs. Stage 4	41	0.287	−0.697	(−1.985; 0.590)
LH				
Tanner Stage 2 vs. Stage 1	172	<0.001	0.545	(0.309; 0.781)
Tanner Stage 3 vs. Stage 2	111	<0.001	1.163	(0.843; 1.484)
Tanner Stage 4 vs. Stage 3	71	<0.001	0.963	(0.545; 1.380)
Tanner Stage 5 vs. Stage 4	40	0.805	0.080	(−0.558; 0.718)
Oestradiol				
Tanner Stage 2 vs. Stage 1	173	0.07	6.120	(−0.494; 12.733)
Tanner Stage 3 vs. Stage 2	112	<0.001	19.993	(10.968; 29.018)
Tanner Stage 4 vs. Stage 3	72	0.022	13.621	(2.011; 25.230)
Tanner Stage 5 vs. Stage 4	41	0.006	25.320	(7.430; 43.210)
Testosterone				
Tanner Stage 2 vs. Stage 1	174	0.186	0.548	(−0.266; 1.363)
Tanner Stage 3 vs. Stage 2	111	<0.001	5.237	(4.127; 6.347)
Tanner Stage 4 vs. Stage 3	72	<0.001	3.347	(1.922; 4.772)
Tanner Stage 5 vs. Stage 4	41	<0.001	4.621	(2.425; 6.818)
SHBG				
Tanner Stage 2 vs. Stage 1	172	0.008	−13.531	(−23.454; −3.608)
Tanner Stage 3 vs. Stage 2	111	<0.001	−38.669	(−52.146; −25.191)
Tanner Stage 4 vs. Stage 3	71	0.009	−23.341	(−40.904; −5.778)
Tanner Stage 5 vs. Stage 4	40	0.999	0.023	(−26.805; 26.850)

**Table 6:** Levels of gonadotropins during sexual development in this study compared with other published studies.

Study	Country	Study Type	Summary Statistics	FSH (IU/L)					LH(IU/L)				
				Tanner Stage 1	Tanner Stage 2	Tanner Stage 3	Tanner Stage 4	Tanner Stage 5	Tanner Stage 1	Tanner Stage 2	Tanner Stage 3	Tanner Stage 4	Tanner Stage 5
Current study	South Africa	C-S	Median	0.9	1.9 *	3.1 *	5.0 *	3.4	0.05	0.7 *	1.65 *	3.0 *	2.8
			IQR	0.5–1.5	1.3–3.1	2.3–4.9	3.2–7.5	3.2–7.0	0.05–0.2	0.2–1.0	1.3–2.1	1.8–4.2	1.9–3.8
			*N*	98	72	38	34	7	99	73	38	33	7
Andersson et al. [15]	Denmark	C-S	Median	0.85	1.95 *	3.50 *	3.61	3.1	0.08	0.88 *	2.03 *	2.89 *	3.40 *
			90% PI	0.25–2.55	0.07–4.39	0.94–9.68	1.98–6.88	1.38–7.52	0.05–0.99	0.11–2.97	0.51–5.42	1.11–5.89	1.53–6.33
			*N*	154	47	27	31	128	154	47	26	31	129
Raivio et al. [17]	Finland	L	Mean	1.8	1.8	1.9	2.9	3	0.3	1.1	1.4	2	2.7
			±SD	1.2–2.6	1.2–2.8	1.2–3.1	1.7–4.9	1.8–4.9	0.1–0.8	0.5–2.3	0.8–2.5	1.3–3.0	1.6–4.5
			*N*	16	37	37	37		16	37	37	37	
Crofton et al. [18]	Ireland	L	Median	1.2	2.2	2.7 *	3.5	4.2					
			IQR	1.0–2.1	1.6–2.9	1.9–3.3	2.7–5.0	3.7–5.1					
			*N*	90	38	39	18	10					
Chada et al. [19]	Czech Republic	C-S	Mean	0.42	0.85	1.46	2.3		0.21	0.61	1.23	3.17	
			Min–Max	0.20–0.58	0.21–1.48	0.49–2.63	0.93–3.65		0.10–0.44	0.12–1.43	0.45–2.39	1.05–5.54	
			*N*	11	8	12	10		11	8	12	10	

L: longitudinal; C-S: cross-sectional; IQR: interquartile range; *N*: number of boys; PI: predict interval; SD: standard deviation; *: Significance is indicated when the median is statistically significant different from the median of the preceding stage of puberty (*p* < 0.05). FSH and LH were measure by a time-resolved immunofluorometric assay in the Danish and Finnish study, chemiluminescent microparticle immunoassay (CMIA) in the Czech study, and immunoradiometric assay in the Irish study. In the Danish study, a testicular volume greater than 3 mL was taken as a sign of the onset of puberty; the Czech study used Tanner Stage 1 as onset of puberty and in the Irish study Tanner Stage 2 was used as onset of puberty. The onset of puberty was not used in the Finnish study. Adult reference range used in study laboratory for FSH: 1.5–12.4 IU/L and for LH: 1.7–8.6 IU/L.

**Table 7:** Levels of sex steroids during sexual development in this study compared with other published studies.

Study	Country	Study Type	Summary Statistics	Testosterone (nmol/L)					Oestradiol (pmol/L)				
				Tanner Stage 1	Tanner Stage 2	Tanner Stage 3	Tanner Stage 4	Tanner Stage 5	Tanner Stage 1	Tanner Stage 2	Tanner Stage 3	Tanner Stage 4	Tanner Stage 5
Current study	South Africa	C-S	Median	0.05	0.2	3.9 *	10.5 *	12.9 *	31.5	40.5	56.5 *	79.4 *	100.9 *
			IQR	0.05–0.1	0.05–0.6	1.6–8.4	5.4–13.6	10–18.9	23.1–45.5	29.7–50.4	46.1–72.2	61.5–88.5	87.2–115.2
			*N*	101	73	38	34	7	99	74	38	34	7
Andersson et al. [15]	Denmark	C-S	Median	0.2	1.9 *	8.4 *	17.2 *	21.0 *	18	21.0 *	36.0 *	59.0 *	71.0 *
			90% PI	0.2–0.9	0.2–13.4	0.9–21.2	7.7–26.5	11.3–32.3	18.0–34.0	18.0–45.0	18.0–85.0	29.0–102.0	44.0–117.0
			*N*	155	47	27	31	130	154	46	25	31	129
Raivio et al. [17]	Finland	L	Mean	0.4	1.4	3.9	18.1	35.4					
			±SD	0.2–0.9	0.6–3.4	1.6–9.4	8.0–41.0	23.0–55.0					
			*N*	16	37	37	37						
Crofton et al. [18]	Ireland	L	Median	0.3	0.9 *	6.2 *	15.5	18.4					
			IQR	0.3–0.5	0.5–1.7	3.0–11.2	10.2–21.0	15.8–20.0					
			*N*	90	38	39	18	10					
Chada et al. [19]	Czech Republic	C-S	Mean	0.27	0.97	3.73	12.74						
			Min–Max	0.07–0.55	0.44–1.85	1.52–7.21	8.9–18.1						
			*N*	11	8	12	10						

L, longitudinal; C-S, cross-sectional; IQR, interquartile range; *N*, number of boys; PI, predict interval; SD, standard deviation; *: Significance is indicated when the median is statistically significant different from the median of the preceding stage of puberty (*p* < 0.05). Testosterol levels were measured by radioimmunoassay (RIA) for all overseas studies. Oestradiol levels were measured by radioimmunoassay (RIA) in the Danish study. Adult reference range used in study laboratory for testosterone: 8.4–28.8 nmol/L and for oestradiol: 28–156 pmol/L.
